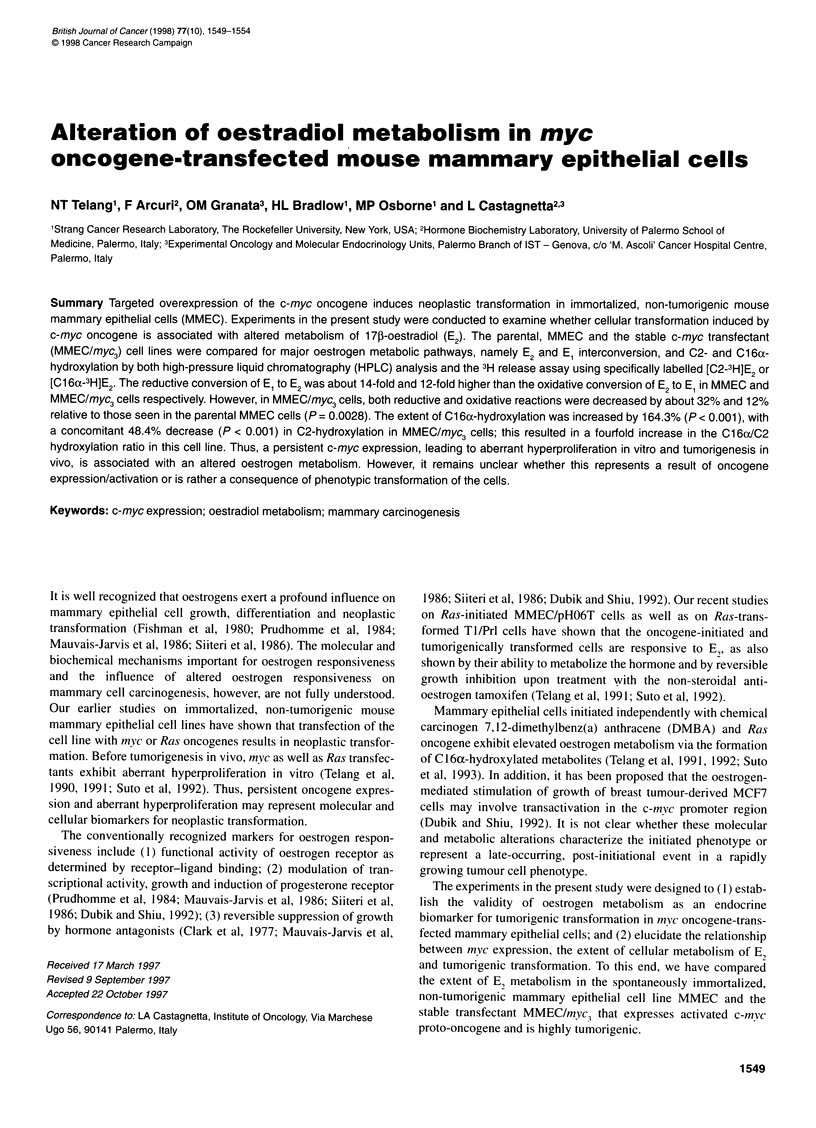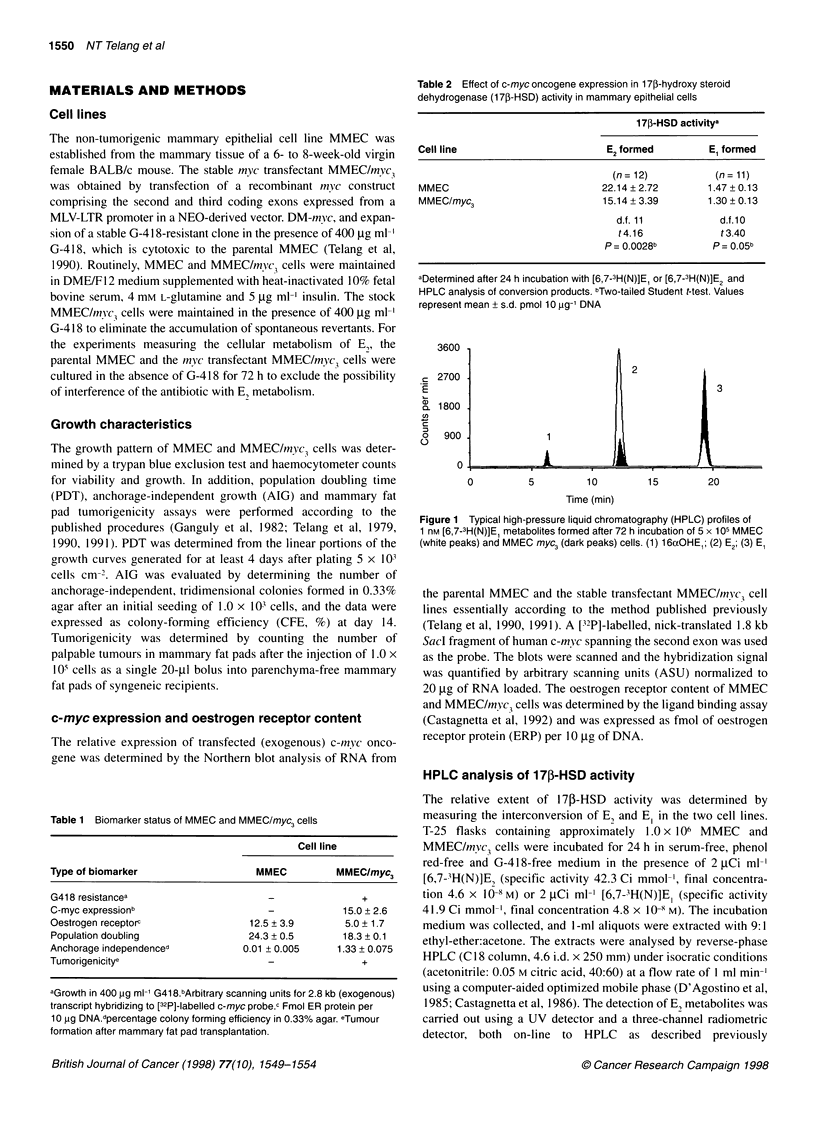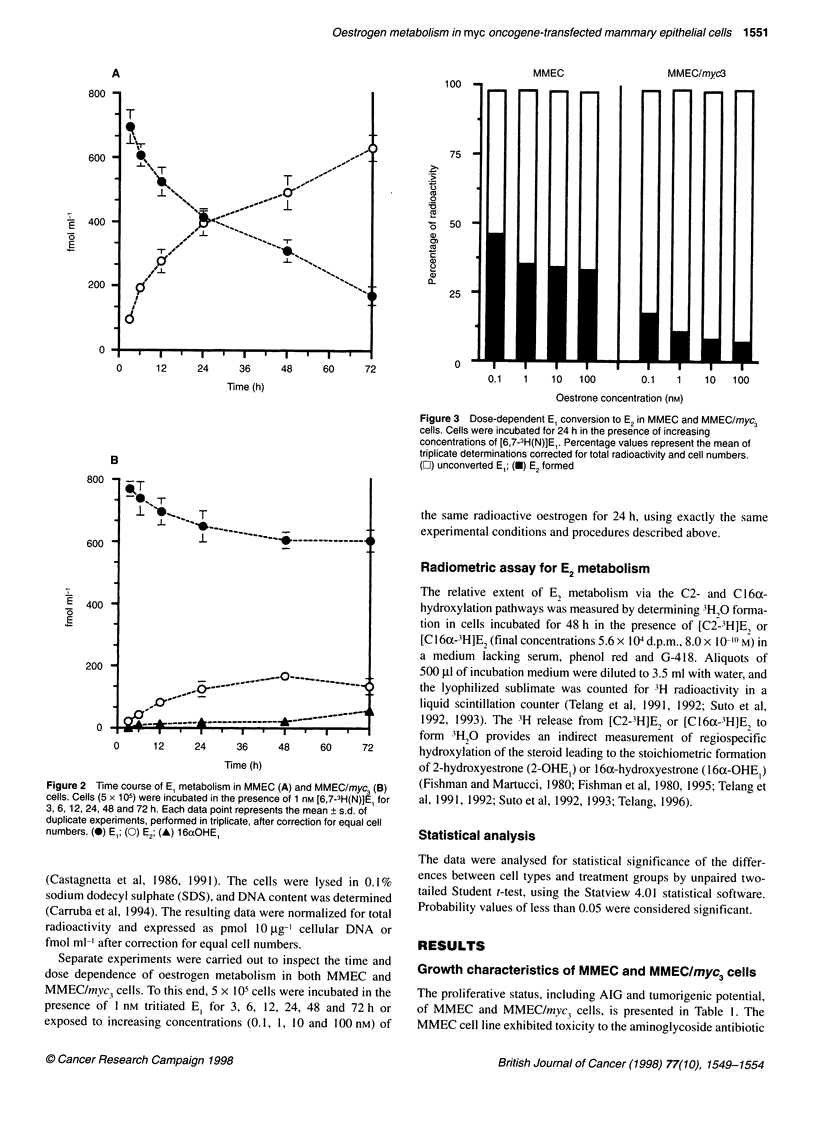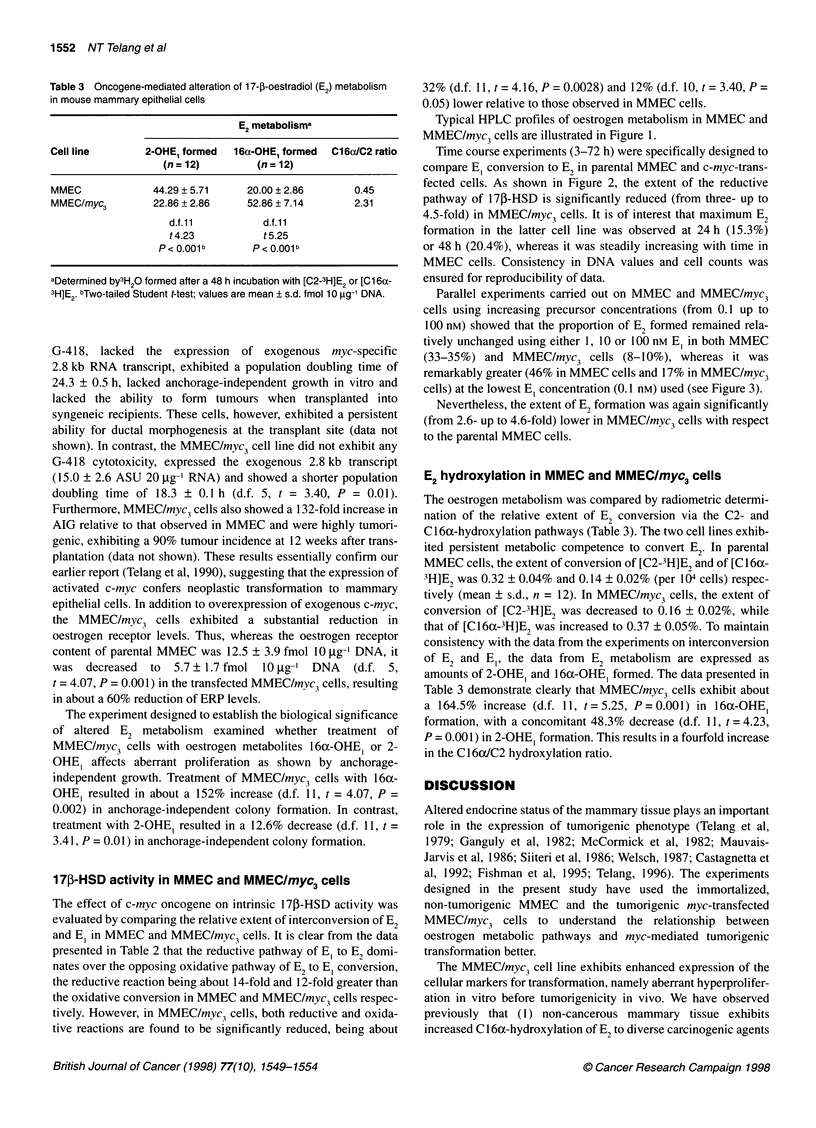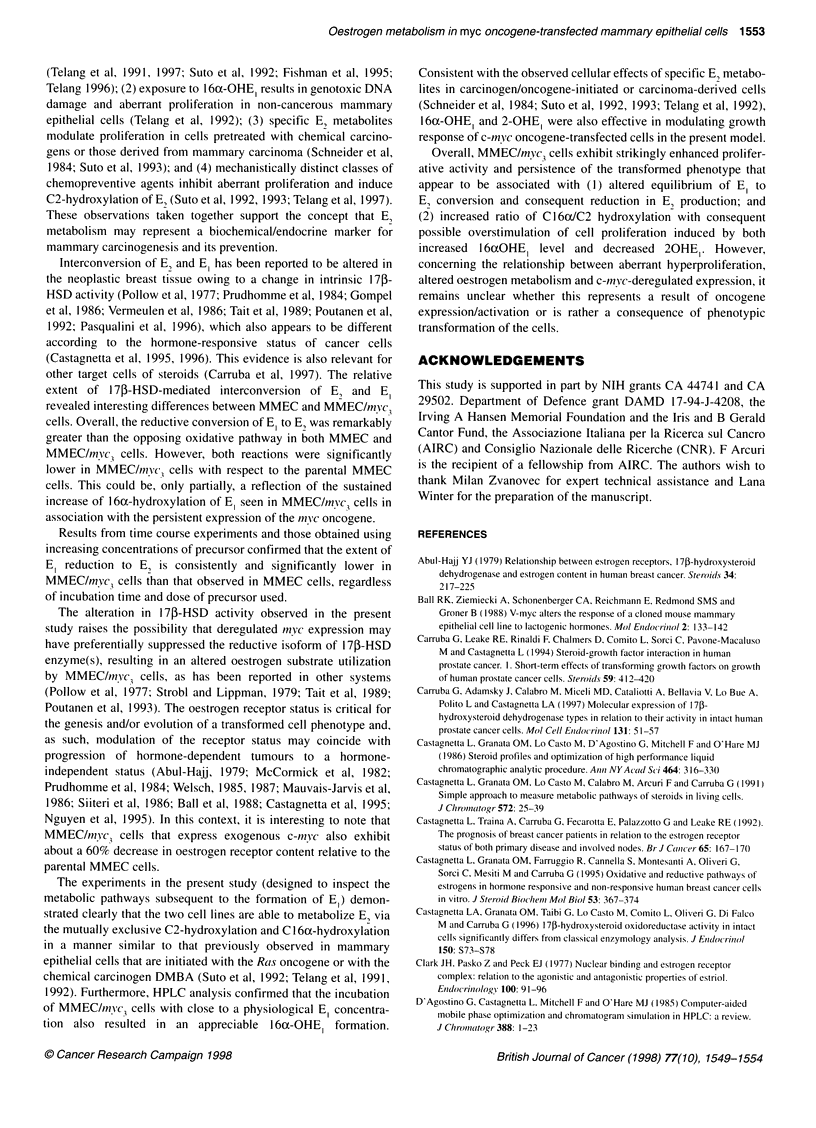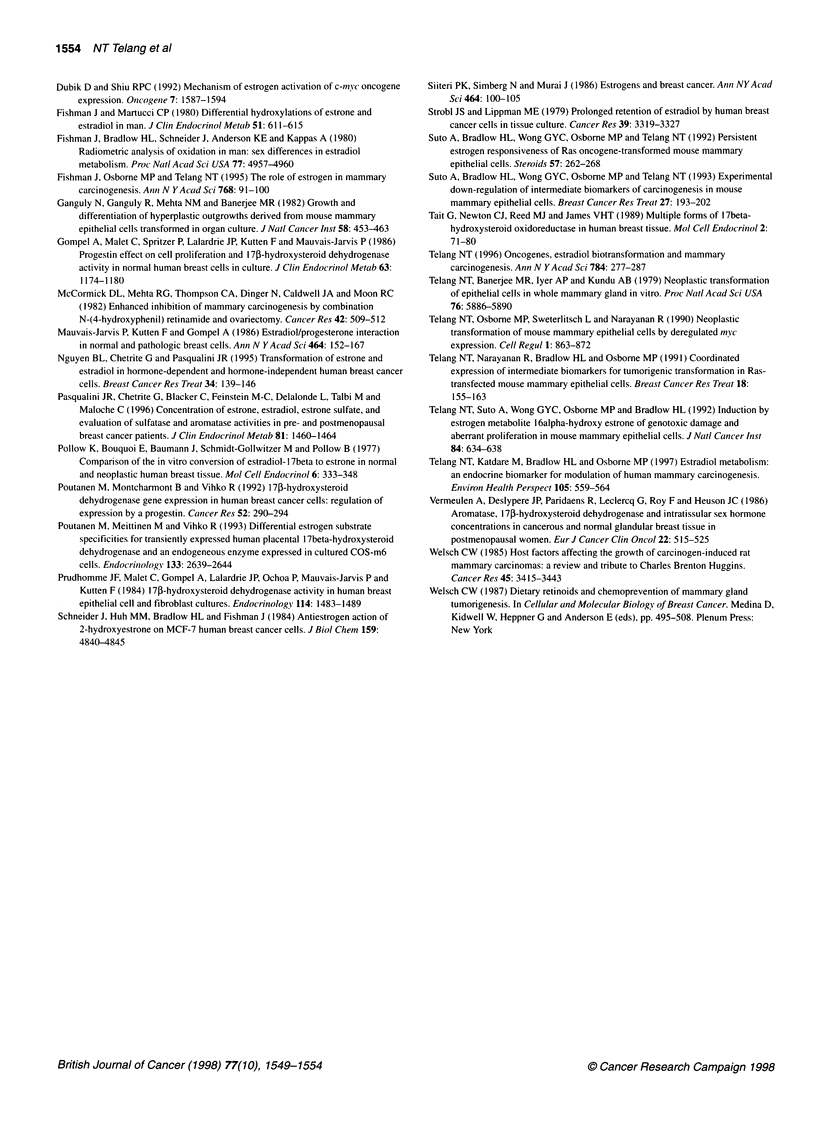# Alteration of oestradiol metabolism in myc oncogene-transfected mouse mammary epithelial cells.

**DOI:** 10.1038/bjc.1998.255

**Published:** 1998-05

**Authors:** N. T. Telang, F. Arcuri, O. M. Granata, H. L. Bradlow, M. P. Osborne, L. Castagnetta

**Affiliations:** Strang Cancer Research Laboratory, The Rockefeller University, New York, USA.

## Abstract

Targeted overexpression of the c-myc oncogene induces neoplastic transformation in immortalized, non-tumorigenic mouse mammary epithelial cells (MMEC). Experiments in the present study were conducted to examine whether cellular transformation induced by c-myc oncogene is associated with altered metabolism of 17beta-oestradiol (E2). The parental, MMEC and the stable c-myc transfectant (MMEC/myc3) cell lines were compared for major oestrogen metabolic pathways, namely E2 and E1 interconversion, and C2- and C16alpha-hydroxylation by both high-pressure liquid chromatography (HPLC) analysis and the 3H release assay using specifically labelled [C2-3H]E2 or [C16alpha-3H]E2. The reductive conversion of E1 to E2 was about 14-fold and 12-fold higher than the oxidative conversion of E2 to E1 in MMEC and MMEC/myc3 cells respectively. However, in MMEC/myc3 cells, both reductive and oxidative reactions were decreased by about 32% and 12% relative to those seen in the parental MMEC cells (P = 0.0028). The extent of C16alpha-hydroxylation was increased by 164.3% (P < 0.001), with a concomitant 48.4% decrease (P < 0.001) in C2-hydroxylation in MMEC/myc3 cells; this resulted in a fourfold increase in the C16alpha/C2 hydroxylation ratio in this cell line. Thus, a persistent c-myc expression, leading to aberrant hyperproliferation in vitro and tumorigenesis in vivo, is associated with an altered oestrogen metabolism. However, it remains unclear whether this represents a result of oncogene expression/activation or is rather a consequence of phenotypic transformation of the cells.


British Journal of Cancer (1998) 77(10), 1549–1554
© 1998 Cancer Research Campaign

### Alteration of oestradiol metabolism in *myc* oncogene-transfected mouse mammary epithelial cells

**NT Telang[1], F Arcuri[2], OM Granata[3], HL Bradlow[1], MP Osborne[1] and L Castagnetta[2,3]**

[1]Strang Cancer Research Laboratory, The Rockefeller University, New York, USA; [2]Hormone Biochemistry Laboratory, University of Palermo School of Medicine, Palermo, Italy; [3]Experimental Oncology and Molecular Endocrinology Units, Palermo Branch of IST – Genova, c/o 'M. Ascoli' Cancer Hospital Centre, Palermo, Italy

**Summary** Targeted overexpression of the c-*myc* oncogene induces neoplastic transformation in immortalized, non-tumorigenic mouse mammary epithelial cells (MMEC). Experiments in the present study were conducted to examine whether cellular transformation induced by c-*myc* oncogene is associated with altered metabolism of 17β-oestradiol ($E_2$). The parental, MMEC and the stable c-*myc* transfectant (MMEC/*myc*$_3$) cell lines were compared for major oestrogen metabolic pathways, namely $E_2$ and $E_1$ interconversion, and C2- and C16α-hydroxylation by both high-pressure liquid chromatography (HPLC) analysis and the $^3$H release assay using specifically labelled [C2-$^3$H]$E_2$ or [C16α-$^3$H]$E_2$. The reductive conversion of $E_1$ to $E_2$ was about 14-fold and 12-fold higher than the oxidative conversion of $E_2$ to $E_1$ in MMEC and MMEC/*myc*$_3$ cells respectively. However, in MMEC/*myc*$_3$ cells, both reductive and oxidative reactions were decreased by about 32% and 12% relative to those seen in the parental MMEC cells ($P = 0.0028$). The extent of C16α-hydroxylation was increased by 164.3% ($P < 0.001$), with a concomitant 48.4% decrease ($P < 0.001$) in C2-hydroxylation in MMEC/*myc*$_3$ cells; this resulted in a fourfold increase in the C16α/C2 hydroxylation ratio in this cell line. Thus, a persistent c-*myc* expression, leading to aberrant hyperproliferation in vitro and tumorigenesis in vivo, is associated with an altered oestrogen metabolism. However, it remains unclear whether this represents a result of oncogene expression/activation or is rather a consequence of phenotypic transformation of the cells.

**Keywords:** c-*myc* expression; oestradiol metabolism; mammary carcinogenesis

It is well recognized that oestrogens exert a profound influence on mammary epithelial cell growth, differentiation and neoplastic transformation (Fishman et al, 1980; Prudhomme et al, 1984; Mauvais-Jarvis et al, 1986; Siiteri et al, 1986). The molecular and biochemical mechanisms important for oestrogen responsiveness and the influence of altered oestrogen responsiveness on mammary cell carcinogenesis, however, are not fully understood. Our earlier studies on immortalized, non-tumorigenic mouse mammary epithelial cell lines have shown that transfection of the cell line with *myc* or *Ras* oncogenes results in neoplastic transformation. Before tumorigenesis in vivo, *myc* as well as *Ras* transfectants exhibit aberrant hyperproliferation in vitro (Telang et al, 1990, 1991; Suto et al, 1992). Thus, persistent oncogene expression and aberrant hyperproliferation may represent molecular and cellular biomarkers for neoplastic transformation.

The conventionally recognized markers for oestrogen responsiveness include (1) functional activity of oestrogen receptor as determined by receptor–ligand binding; (2) modulation of transcriptional activity, growth and induction of progesterone receptor (Prudhomme et al, 1984; Mauvais-Jarvis et al, 1986; Siiteri et al, 1986; Dubik and Shiu, 1992); (3) reversible suppression of growth by hormone antagonists (Clark et al, 1977; Mauvais-Jarvis et al, 1986; Siiteri et al, 1986; Dubik and Shiu, 1992). Our recent studies on *Ras*-initiated MMEC/pH06T cells as well as on *Ras*-transformed T1/Prl cells have shown that the oncogene-initiated and tumorigenically transformed cells are responsive to $E_2$, as also shown by their ability to metabolize the hormone and by reversible growth inhibition upon treatment with the non-steroidal anti-oestrogen tamoxifen (Telang et al, 1991; Suto et al, 1992).

Mammary epithelial cells initiated independently with chemical carcinogen 7,12-dimethylbenz(a) anthracene (DMBA) and *Ras* oncogene exhibit elevated oestrogen metabolism via the formation of C16α-hydroxylated metabolites (Telang et al, 1991, 1992; Suto et al, 1993). In addition, it has been proposed that the oestrogen-mediated stimulation of growth of breast tumour-derived MCF7 cells may involve transactivation in the c-*myc* promoter region (Dubik and Shiu, 1992). It is not clear whether these molecular and metabolic alterations characterize the initiated phenotype or represent a late-occurring, post-initiational event in a rapidly growing tumour cell phenotype.

The experiments in the present study were designed to (1) establish the validity of oestrogen metabolism as an endocrine biomarker for tumorigenic transformation in *myc* oncogene-transfected mammary epithelial cells; and (2) elucidate the relationship between *myc* expression, the extent of cellular metabolism of $E_2$ and tumorigenic transformation. To this end, we have compared the extent of $E_2$ metabolism in the spontaneously immortalized, non-tumorigenic mammary epithelial cell line MMEC and the stable transfectant MMEC/*myc*$_3$ that expresses activated c-*myc* proto-oncogene and is highly tumorigenic.

*Received 17 March 1997*
*Revised 9 September 1997*
*Accepted 22 October 1997*

*Correspondence to:* LA Castagnetta, Institute of Oncology, Via Marchese Ugo 56, 90141 Palermo, Italy

### MATERIALS AND METHODS

### Cell lines

The non-tumorigenic mammary epithelial cell line MMEC was established from the mammary tissue of a 6- to 8-week-old virgin female BALB/c mouse. The stable *myc* transfectant MMEC/*myc*$_3$ was obtained by transfection of a recombinant *myc* construct comprising the second and third coding exons expressed from a MLV-LTR promoter in a NEO-derived vector. DM-*myc*, and expansion of a stable G-418-resistant clone in the presence of 400 μg ml$^{-1}$ G-418, which is cytotoxic to the parental MMEC (Telang et al, 1990). Routinely, MMEC and MMEC/*myc*$_3$ cells were maintained in DME/F12 medium supplemented with heat-inactivated 10% fetal bovine serum, 4 mM L-glutamine and 5 μg ml$^{-1}$ insulin. The stock MMEC/*myc*$_3$ cells were maintained in the presence of 400 μg ml$^{-1}$ G-418 to eliminate the accumulation of spontaneous revertants. For the experiments measuring the cellular metabolism of $E_2$, the parental MMEC and the *myc* transfectant MMEC/*myc*$_3$ cells were cultured in the absence of G-418 for 72 h to exclude the possibility of interference of the antibiotic with $E_2$ metabolism.

### Growth characteristics

The growth pattern of MMEC and MMEC/*myc*$_3$ cells was determined by a trypan blue exclusion test and haemocytometer counts for viability and growth. In addition, population doubling time (PDT), anchorage-independent growth (AIG) and mammary fat pad tumorigenicity assays were performed according to the published procedures (Ganguly et al, 1982; Telang et al, 1979, 1990, 1991). PDT was determined from the linear portions of the growth curves generated for at least 4 days after plating 5 × 10$^3$ cells cm$^{-2}$. AIG was evaluated by determining the number of anchorage-independent, tridimensional colonies formed in 0.33% agar after an initial seeding of 1.0 × 10$^3$ cells, and the data were expressed as colony-forming efficiency (CFE, %) at day 14. Tumorigenicity was determined by counting the number of palpable tumours in mammary fat pads after the injection of 1.0 × 10$^5$ cells as a single 20-μl bolus into parenchyma-free mammary fat pads of syngeneic recipients.

### c-*myc* expression and oestrogen receptor content

The relative expression of transfected (exogenous) c-*myc* oncogene was determined by the Northern blot analysis of RNA from the parental MMEC and the stable transfectant MMEC/*myc*$_3$ cell lines essentially according to the method published previously (Telang et al, 1990, 1991). A [$^{32}$P]-labelled, nick-translated 1.8 kb *Sac*I fragment of human c-*myc* spanning the second exon was used as the probe. The blots were scanned and the hybridization signal was quantified by arbitrary scanning units (ASU) normalized to 20 μg of RNA loaded. The oestrogen receptor content of MMEC and MMEC/*myc*$_3$ cells was determined by the ligand binding assay (Castagnetta et al, 1992) and was expressed as fmol of oestrogen receptor protein (ERP) per 10 μg of DNA.

**Table 1** Biomarker status of MMEC and MMEC/*myc*$_3$ cells

| Type of biomarker | Cell line: MMEC | Cell line: MMEC/*myc*$_3$ |
|---|---|---|
| G418 resistance[a] | – | + |
| C-myc expression[b] | – | 15.0 ± 2.6 |
| Oestrogen receptor[c] | 12.5 ± 3.9 | 5.0 ± 1.7 |
| Population doubling | 24.3 ± 0.5 | 18.3 ± 0.1 |
| Anchorage independence[d] | 0.01 ± 0.005 | 1.33 ± 0.075 |
| Tumorigenicity[e] | – | + |

[a]Growth in 400 μg ml$^{-1}$ G418.[b]Arbitrary scanning units for 2.8 kb (exogenous) transcript hybridizing to [$^{32}$P]-labelled c-*myc* probe.[c] Fmol ER protein per 10 μg DNA.[d]percentage colony forming efficiency in 0.33% agar. [e]Tumour formation after mammary fat pad transplantation.

**Table 2** Effect of c-*myc* oncogene expression in 17β-hydroxy steroid dehydrogenase (17β-HSD) activity in mammary epithelial cells

| Cell line | 17β-HSD activity[a]: $E_2$ formed | 17β-HSD activity[a]: $E_1$ formed |
|---|---|---|
| | ($n$ = 12) | ($n$ = 11) |
| MMEC | 22.14 ± 2.72 | 1.47 ± 0.13 |
| MMEC/*myc*$_3$ | 15.14 ± 3.39 | 1.30 ± 0.13 |
| | d.f. 11 | d.f.10 |
| | $t$ 4.16 | $t$ 3.40 |
| | $P$ = 0.0028[b] | $P$ = 0.05[b] |

[a]Determined after 24 h incubation with [6,7-$^3$H(N)]$E_1$ or [6,7-$^3$H(N)]$E_2$ and HPLC analysis of conversion products. [b]Two-tailed Student $t$-test. Values represent mean ± s.d. pmol 10 μg$^{-1}$ DNA

**Figure 1** Typical high-pressure liquid chromatography (HPLC) profiles of 1 nM [6,7-$^3$H(N)]$E_1$ metabolites formed after 72 h incubation of 5 × 10$^5$ MMEC (white peaks) and MMEC *myc*$_3$ (dark peaks) cells. (1) 16αOHE$_1$; (2) $E_2$; (3) $E_1$

### HPLC analysis of 17β-HSD activity

The relative extent of 17β-HSD activity was determined by measuring the interconversion of $E_2$ and $E_1$ in the two cell lines. T-25 flasks containing approximately 1.0 × 10$^6$ MMEC and MMEC/*myc*$_3$ cells were incubated for 24 h in serum-free, phenol red-free and G-418-free medium in the presence of 2 μCi ml$^{-1}$ [6,7-$^3$H(N)]$E_2$ (specific activity 42.3 Ci mmol$^{-1}$, final concentration 4.6 × 10$^{-8}$ M) or 2 μCi ml$^{-1}$ [6,7-$^3$H(N)]$E_1$ (specific activity 41.9 Ci mmol$^{-1}$, final concentration 4.8 × 10$^{-8}$ M). The incubation medium was collected, and 1-ml aliquots were extracted with 9:1 ethyl-ether:acetone. The extracts were analysed by reverse-phase HPLC (C18 column, 4.6 i.d. × 250 mm) under isocratic conditions (acetonitrile: 0.05 M citric acid, 40:60) at a flow rate of 1 ml min$^{-1}$ using a computer-aided optimized mobile phase (D'Agostino et al, 1985; Castagnetta et al, 1986). The detection of $E_2$ metabolites was carried out using a UV detector and a three-channel radiometric detector, both on-line to HPLC as described previously

© Cancer Research Campaign 1998

**Figure 2** Time course of $E_1$ metabolism in MMEC (**A**) and MMEC/*myc*$_3$ (**B**) cells. Cells ($5 \times 10^5$) were incubated in the presence of 1 nM [6,7-$^3$H(N)]$E_1$ for 3, 6, 12, 24, 48 and 72 h. Each data point represents the mean ± s.d. of duplicate experiments, performed in triplicate, after correction for equal cell numbers. (●) $E_1$; (○) $E_2$; (▲) 16αOHE$_1$

**Figure 3** Dose-dependent $E_1$ conversion to $E_2$ in MMEC and MMEC/*myc*$_3$ cells. Cells were incubated for 24 h in the presence of increasing concentrations of [6,7-$^3$H(N)]$E_1$. Percentage values represent the mean of triplicate determinations corrected for total radioactivity and cell numbers. (□) unconverted $E_1$; (■) $E_2$ formed

(Castagnetta et al, 1986, 1991). The cells were lysed in 0.1% sodium dodecyl sulphate (SDS), and DNA content was determined (Carruba et al, 1994). The resulting data were normalized for total radioactivity and expressed as pmol 10 μg$^{-1}$ cellular DNA or fmol ml$^{-1}$ after correction for equal cell numbers.

Separate experiments were carried out to inspect the time and dose dependence of oestrogen metabolism in both MMEC and MMEC/*myc*$_3$ cells. To this end, $5 \times 10^5$ cells were incubated in the presence of 1 nM tritiated $E_1$ for 3, 6, 12, 24, 48 and 72 h or exposed to increasing concentrations (0.1, 1, 10 and 100 nM) of the same radioactive oestrogen for 24 h, using exactly the same experimental conditions and procedures described above.

### Radiometric assay for $E_2$ metabolism

The relative extent of $E_2$ metabolism via the C2- and C16α-hydroxylation pathways was measured by determining $^3H_2O$ formation in cells incubated for 48 h in the presence of [C2-$^3$H]$E_2$ or [C16α-$^3$H]$E_2$ (final concentrations $5.6 \times 10^4$ d.p.m., $8.0 \times 10^{-10}$ M) in a medium lacking serum, phenol red and G-418. Aliquots of 500 μl of incubation medium were diluted to 3.5 ml with water, and the lyophilized sublimate was counted for $^3$H radioactivity in a liquid scintillation counter (Telang et al, 1991, 1992; Suto et al, 1992, 1993). The $^3$H release from [C2-$^3$H]$E_2$ or [C16α-$^3$H]$E_2$ to form $^3H_2O$ provides an indirect measurement of regiospecific hydroxylation of the steroid leading to the stoichiometric formation of 2-hydroxyestrone (2-OHE$_1$) or 16α-hydroxyestrone (16α-OHE$_1$) (Fishman and Martucci, 1980; Fishman et al, 1980, 1995; Telang et al, 1991, 1992; Suto et al, 1992, 1993; Telang, 1996).

### Statistical analysis

The data were analysed for statistical significance of the differences between cell types and treatment groups by unpaired two-tailed Student *t*-test, using the Statview 4.01 statistical software. Probability values of less than 0.05 were considered significant.

### RESULTS

### Growth characteristics of MMEC and MMEC/*myc*$_3$ cells

The proliferative status, including AIG and tumorigenic potential, of MMEC and MMEC/*myc*$_3$ cells, is presented in Table 1. The MMEC cell line exhibited toxicity to the aminoglycoside antibiotic

© *Cancer Research Campaign 1998*

**Table 3** Oncogene-mediated alteration of 17-β-oestradiol ($E_2$) metabolism in mouse mammary epithelial cells

| Cell line | $E_2$ metabolism[a] | | |
|---|---|---|---|
| | 2-$OHE_1$ formed ($n$ = 12) | 16α-$OHE_1$ formed ($n$ = 12) | C16α/C2 ratio |
| MMEC | 44.29 ± 5.71 | 20.00 ± 2.86 | 0.45 |
| MMEC/*myc*$_3$ | 22.86 ± 2.86 | 52.86 ± 7.14 | 2.31 |
| | d.f.11 | d.f.11 | |
| | $t$4.23 | $t$5.25 | |
| | $P$ < 0.001[b] | $P$ < 0.001[b] | |

[a]Determined by $^3H_2O$ formed after a 48 h incubation with [C2-$^3$H]$E_2$ or [C16α-$^3$H]$E_2$. [b]Two-tailed Student $t$-test; values are mean ± s.d. fmol 10 μg$^{-1}$ DNA.

G-418, lacked the expression of exogenous *myc*-specific 2.8 kb RNA transcript, exhibited a population doubling time of 24.3 ± 0.5 h, lacked anchorage-independent growth in vitro and lacked the ability to form tumours when transplanted into syngeneic recipients. These cells, however, exhibited a persistent ability for ductal morphogenesis at the transplant site (data not shown). In contrast, the MMEC/*myc*$_3$ cell line did not exhibit any G-418 cytotoxicity, expressed the exogenous 2.8 kb transcript (15.0 ± 2.6 ASU 20 μg$^{-1}$ RNA) and showed a shorter population doubling time of 18.3 ± 0.1 h (d.f. 5, $t$ = 3.40, $P$ = 0.01). Furthermore, MMEC/*myc*$_3$ cells also showed a 132-fold increase in AIG relative to that observed in MMEC and were highly tumorigenic, exhibiting a 90% tumour incidence at 12 weeks after transplantation (data not shown). These results essentially confirm our earlier report (Telang et al, 1990), suggesting that the expression of activated c-*myc* confers neoplastic transformation to mammary epithelial cells. In addition to overexpression of exogenous c-*myc*, the MMEC/*myc*$_3$ cells exhibited a substantial reduction in oestrogen receptor levels. Thus, whereas the oestrogen receptor content of parental MMEC was 12.5 ± 3.9 fmol 10 μg$^{-1}$ DNA, it was decreased to 5.7 ± 1.7 fmol 10 μg$^{-1}$ DNA (d.f. 5, $t$ = 4.07, $P$ = 0.001) in the transfected MMEC/*myc*$_3$ cells, resulting in about a 60% reduction of ERP levels.

The experiment designed to establish the biological significance of altered $E_2$ metabolism examined whether treatment of MMEC/*myc*$_3$ cells with oestrogen metabolites 16α-$OHE_1$ or 2-$OHE_1$ affects aberrant proliferation as shown by anchorage-independent growth. Treatment of MMEC/*myc*$_3$ cells with 16α-$OHE_1$ resulted in about a 152% increase (d.f. 11, $t$ = 4.07, $P$ = 0.002) in anchorage-independent colony formation. In contrast, treatment with 2-$OHE_1$ resulted in a 12.6% decrease (d.f. 11, $t$ = 3.41, $P$ = 0.01) in anchorage-independent colony formation.

### 17β-HSD activity in MMEC and MMEC/*myc*$_3$ cells

The effect of c-*myc* oncogene on intrinsic 17β-HSD activity was evaluated by comparing the relative extent of interconversion of $E_2$ and $E_1$ in MMEC and MMEC/*myc*$_3$ cells. It is clear from the data presented in Table 2 that the reductive pathway of $E_1$ to $E_2$ dominates over the opposing oxidative pathway of $E_2$ to $E_1$ conversion, the reductive reaction being about 14-fold and 12-fold greater than the oxidative conversion in MMEC and MMEC/*myc*$_3$ cells respectively. However, in MMEC/*myc*$_3$ cells, both reductive and oxidative reactions are found to be significantly reduced, being about 32% (d.f. 11, $t$ = 4.16, $P$ = 0.0028) and 12% (d.f. 10, $t$ = 3.40, $P$ = 0.05) lower relative to those observed in MMEC cells.

Typical HPLC profiles of oestrogen metabolism in MMEC and MMEC/*myc*$_3$ cells are illustrated in Figure 1.

Time course experiments (3–72 h) were specifically designed to compare $E_1$ conversion to $E_2$ in parental MMEC and c-*myc*-transfected cells. As shown in Figure 2, the extent of the reductive pathway of 17β-HSD is significantly reduced (from three- up to 4.5-fold) in MMEC/*myc*$_3$ cells. It is of interest that maximum $E_2$ formation in the latter cell line was observed at 24 h (15.3%) or 48 h (20.4%), whereas it was steadily increasing with time in MMEC cells. Consistency in DNA values and cell counts was ensured for reproducibility of data.

Parallel experiments carried out on MMEC and MMEC/*myc*$_3$ cells using increasing precursor concentrations (from 0.1 up to 100 nM) showed that the proportion of $E_2$ formed remained relatively unchanged using either 1, 10 or 100 nM $E_1$ in both MMEC (33–35%) and MMEC/*myc*$_3$ cells (8–10%), whereas it was remarkably greater (46% in MMEC cells and 17% in MMEC/*myc*$_3$ cells) at the lowest $E_1$ concentration (0.1 nM) used (see Figure 3).

Nevertheless, the extent of $E_2$ formation was again significantly (from 2.6- up to 4.6-fold) lower in MMEC/*myc*$_3$ cells with respect to the parental MMEC cells.

### $E_2$ hydroxylation in MMEC and MMEC/*myc*$_3$ cells

The oestrogen metabolism was compared by radiometric determination of the relative extent of $E_2$ conversion via the C2- and C16α-hydroxylation pathways (Table 3). The two cell lines exhibited persistent metabolic competence to convert $E_2$. In parental MMEC cells, the extent of conversion of [C2-$^3$H]$E_2$ and of [C16α-$^3$H]$E_2$ was 0.32 ± 0.04% and 0.14 ± 0.02% (per $10^4$ cells) respectively (mean ± s.d., $n$ = 12). In MMEC/*myc*$_3$ cells, the extent of conversion of [C2-$^3$H]$E_2$ was decreased to 0.16 ± 0.02%, while that of [C16α-$^3$H]$E_2$ was increased to 0.37 ± 0.05%. To maintain consistency with the data from the experiments on interconversion of $E_2$ and $E_1$, the data from $E_2$ metabolism are expressed as amounts of 2-$OHE_1$ and 16α-$OHE_1$ formed. The data presented in Table 3 demonstrate clearly that MMEC/*myc*$_3$ cells exhibit about a 164.5% increase (d.f. 11, $t$ = 5.25, $P$ = 0.001) in 16α-$OHE_1$ formation, with a concomitant 48.3% decrease (d.f. 11, $t$ = 4.23, $P$ = 0.001) in 2-$OHE_1$ formation. This results in a fourfold increase in the C16α/C2 hydroxylation ratio.

### DISCUSSION

Altered endocrine status of the mammary tissue plays an important role in the expression of tumorigenic phenotype (Telang et al, 1979; Ganguly et al, 1982; McCormick et al, 1982; Mauvais-Jarvis et al, 1986; Siiteri et al, 1986; Welsch, 1987; Castagnetta et al, 1992; Fishman et al, 1995; Telang, 1996). The experiments designed in the present study have used the immortalized, non-tumorigenic MMEC and the tumorigenic *myc*-transfected MMEC/*myc*$_3$ cells to understand the relationship between oestrogen metabolic pathways and *myc*-mediated tumorigenic transformation better.

The MMEC/*myc*$_3$ cell line exhibits enhanced expression of the cellular markers for transformation, namely aberrant hyperproliferation in vitro before tumorigenicity in vivo. We have observed previously that (1) non-cancerous mammary tissue exhibits increased C16α-hydroxylation of $E_2$ to diverse carcinogenic agents

 © Cancer Research Campaign 1998

(Telang et al, 1991, 1997; Suto et al, 1992; Fishman et al, 1995; Telang 1996); (2) exposure to 16α-OHE$_1$ results in genotoxic DNA damage and aberrant proliferation in non-cancerous mammary epithelial cells (Telang et al, 1992); (3) specific E$_2$ metabolites modulate proliferation in cells pretreated with chemical carcinogens or those derived from mammary carcinoma (Schneider et al, 1984; Suto et al, 1993); and (4) mechanistically distinct classes of chemopreventive agents inhibit aberrant proliferation and induce C2-hydroxylation of E$_2$ (Suto et al, 1992, 1993; Telang et al, 1997). These observations taken together support the concept that E$_2$ metabolism may represent a biochemical/endocrine marker for mammary carcinogenesis and its prevention.

Interconversion of E$_2$ and E$_1$ has been reported to be altered in the neoplastic breast tissue owing to a change in intrinsic 17β-HSD activity (Pollow et al, 1977; Prudhomme et al, 1984; Gompel et al, 1986; Vermeulen et al, 1986; Tait et al, 1989; Poutanen et al, 1992; Pasqualini et al, 1996), which also appears to be different according to the hormone-responsive status of cancer cells (Castagnetta et al, 1995, 1996). This evidence is also relevant for other target cells of steroids (Carruba et al, 1997). The relative extent of 17β-HSD-mediated interconversion of E$_2$ and E$_1$ revealed interesting differences between MMEC and MMEC/*myc*$_3$ cells. Overall, the reductive conversion of E$_1$ to E$_2$ was remarkably greater than the opposing oxidative pathway in both MMEC and MMEC/*myc*$_3$ cells. However, both reactions were significantly lower in MMEC/*myc*$_3$ cells with respect to the parental MMEC cells. This could be, only partially, a reflection of the sustained increase of 16α-hydroxylation of E$_1$ seen in MMEC/*myc*$_3$ cells in association with the persistent expression of the *myc* oncogene.

Results from time course experiments and those obtained using increasing concentrations of precursor confirmed that the extent of E$_1$ reduction to E$_2$ is consistently and significantly lower in MMEC/*myc*$_3$ cells than that observed in MMEC cells, regardless of incubation time and dose of precursor used.

The alteration in 17β-HSD activity observed in the present study raises the possibility that deregulated *myc* expression may have preferentially suppressed the reductive isoform of 17β-HSD enzyme(s), resulting in an altered oestrogen substrate utilization by MMEC/*myc*$_3$ cells, as has been reported in other systems (Pollow et al, 1977; Strobl and Lippman, 1979; Tait et al, 1989; Poutanen et al, 1993). The oestrogen receptor status is critical for the genesis and/or evolution of a transformed cell phenotype and, as such, modulation of the receptor status may coincide with progression of hormone-dependent tumours to a hormone-independent status (Abul-Hajj, 1979; McCormick et al, 1982; Prudhomme et al, 1984; Welsch, 1985, 1987; Mauvais-Jarvis et al, 1986; Siiteri et al, 1986; Ball et al, 1988; Castagnetta et al, 1995; Nguyen et al, 1995). In this context, it is interesting to note that MMEC/*myc*$_3$ cells that express exogenous c-*myc* also exhibit about a 60% decrease in oestrogen receptor content relative to the parental MMEC cells.

The experiments in the present study (designed to inspect the metabolic pathways subsequent to the formation of E$_1$) demonstrated clearly that the two cell lines are able to metabolize E$_2$ via the mutually exclusive C2-hydroxylation and C16α-hydroxylation in a manner similar to that previously observed in mammary epithelial cells that are initiated with the *Ras* oncogene or with the chemical carcinogen DMBA (Suto et al, 1992; Telang et al, 1991, 1992). Furthermore, HPLC analysis confirmed that the incubation of MMEC/*myc*$_3$ cells with close to a physiological E$_1$ concentration also resulted in an appreciable 16α-OHE$_1$ formation. Consistent with the observed cellular effects of specific E$_2$ metabolites in carcinogen/oncogene-initiated or carcinoma-derived cells (Schneider et al, 1984; Suto et al, 1992, 1993; Telang et al, 1992), 16α-OHE$_1$ and 2-OHE$_1$ were also effective in modulating growth response of c-*myc* oncogene-transfected cells in the present model.

Overall, MMEC/*myc*$_3$ cells exhibit strikingly enhanced proliferative activity and persistence of the transformed phenotype that appear to be associated with (1) altered equilibrium of E$_1$ to E$_2$ conversion and consequent reduction in E$_2$ production; and (2) increased ratio of C16α/C2 hydroxylation with consequent possible overstimulation of cell proliferation induced by both increased 16αOHE$_1$ level and decreased 2OHE$_1$. However, concerning the relationship between aberrant hyperproliferation, altered oestrogen metabolism and c-*myc*-deregulated expression, it remains unclear whether this represents a result of oncogene expression/activation or is rather a consequence of phenotypic transformation of the cells.

### ACKNOWLEDGEMENTS

This study is supported in part by NIH grants CA 44741 and CA 29502. Department of Defence grant DAMD 17-94-J-4208, the Irving A Hansen Memorial Foundation and the Iris and B Gerald Cantor Fund, the Associazione Italiana per la Ricerca sul Cancro (AIRC) and Consiglio Nazionale delle Ricerche (CNR). F Arcuri is the recipient of a fellowship from AIRC. The authors wish to thank Milan Zvanovec for expert technical assistance and Lana Winter for the preparation of the manuscript.

### REFERENCES

Abul-Hajj YJ (1979) Relationship between estrogen receptors, 17β-hydroxysteroid dehydrogenase and estrogen content in human breast cancer. *Steroids* **34**: 217–225

Ball RK, Ziemiecki A, Schonenberger CA, Reichmann E, Redmond SMS and Groner B (1988) V-myc alters the response of a cloned mouse mammary epithelial cell line to lactogenic hormones. *Mol Endocrinol* **2**: 133–142

Carruba G, Leake RE, Rinaldi F, Chalmers D, Comito L, Sorci C, Pavone-Macaluso M and Castagnetta L (1994) Steroid-growth factor interaction in human prostate cancer. 1. Short-term effects of transforming growth factors on growth of human prostate cancer cells. *Steroids* **59**: 412–420

Carruba G, Adamsky J, Calabro M, Miceli MD, Cataliotti A, Bellavia V, Lo Bue A, Polito L and Castagnetta LA (1997) Molecular expression of 17β-hydroxysteroid dehydrogenase types in relation to their activity in intact human prostate cancer cells. *Mol Cell Endocrinol* **131**: 51–57

Castagnetta L, Granata OM, Lo Casto M, D'Agostino G, Mitchell F and O'Hare MJ (1986) Steroid profiles and optimization of high performance liquid chromatographic analytic procedure. *Ann NY Acad Sci* **464**: 316–330

Castagnetta L, Granata OM, Lo Casto M, Calabro M, Arcuri F and Carruba G (1991) Simple approach to measure metabolic pathways of steroids in living cells. *J Chromatogr* **572**: 25–39

Castagnetta L, Traina A, Carruba G, Fecarotta E, Palazzotto G and Leake RE (1992). The prognosis of breast cancer patients in relation to the estrogen receptor status of both primary disease and involved nodes. *Br J Cancer* **65**: 167–170

Castagnetta L, Granata OM, Farruggio R, Cannella S, Montesanti A, Oliveri G, Sorci C, Mesiti M and Carruba G (1995) Oxidative and reductive pathways of estrogens in hormone responsive and non-responsive human breast cancer cells in vitro. *J Steroid Biochem Mol Biol* **53**: 367–374

Castagnetta LA, Granata OM, Taibi G, Lo Casto M, Comito L, Oliveri G, Di Falco M and Carruba G (1996) 17β-hydroxysteroid oxidoreductase activity in intact cells significantly differs from classical enzymology analysis. *J Endocrinol* **150**: S73–S78

Clark JH, Pasko Z and Peck EJ (1977) Nuclear binding and estrogen receptor complex: relation to the agonistic and antagonistic properties of estriol. *Endocrinology* **100**: 91–96

D'Agostino G, Castagnetta L, Mitchell F and O'Hare MJ (1985) Computer-aided mobile phase optimization and chromatogram simulation in HPLC: a review. *J Chromatogr* **388**: 1–23

© Cancer Research Campaign 1998

Dubik D and Shiu RPC (1992) Mechanism of estrogen activation of c-*myc* oncogene expression. *Oncogene* **7**: 1587–1594

Fishman J and Martucci CP (1980) Differential hydroxylations of estrone and estradiol in man. *J Clin Endocrinol Metab* **51**: 611–615

Fishman J, Bradlow HL, Schneider J, Anderson KE and Kappas A (1980) Radiometric analysis of oxidation in man: sex differences in estradiol metabolism. *Proc Natl Acad Sci USA* **77**: 4957–4960

Fishman J, Osborne MP and Telang NT (1995) The role of estrogen in mammary carcinogenesis. *Ann N Y Acad Sci* **768**: 91–100

Ganguly N, Ganguly R, Mehta NM and Banerjee MR (1982) Growth and differentiation of hyperplastic outgrowths derived from mouse mammary epithelial cells transformed in organ culture. *J Natl Cancer Inst* **58**: 453–463

Gompel A, Malet C, Spritzer P, Lalardrie JP, Kutten F and Mauvais-Jarvis P (1986) Progestin effect on cell proliferation and 17β-hydroxysteroid dehydrogenase activity in normal human breast cells in culture. *J Clin Endocrinol Metab* **63**: 1174–1180

McCormick DL, Mehta RG, Thompson CA, Dinger N, Caldwell JA and Moon RC (1982) Enhanced inhibition of mammary carcinogenesis by combination N-(4-hydroxyphenil) retinamide and ovariectomy. *Cancer Res* **42**: 509–512

Mauvais-Jarvis P, Kutten F and Gompel A (1986) Estradiol/progesterone interaction in normal and pathologic breast cells. *Ann N Y Acad Sci* **464**: 152–167

Nguyen BL, Chetrite G and Pasqualini JR (1995) Transformation of estrone and estradiol in hormone-dependent and hormone-independent human breast cancer cells. *Breast Cancer Res Treat* **34**: 139–146

Pasqualini JR, Chetrite G, Blacker C, Feinstein M-C, Delalonde L, Talbi M and Maloche C (1996) Concentration of estrone, estradiol, estrone sulfate, and evaluation of sulfatase and aromatase activities in pre- and postmenopausal breast cancer patients. *J Clin Endocrinol Metab* **81**: 1460–1464

Pollow K, Bouquoi E, Baumann J, Schmidt-Gollwitzer M and Pollow B (1977) Comparison of the in vitro conversion of estradiol-17beta to estrone in normal and neoplastic human breast tissue. *Mol Cell Endocrinol* **6**: 333–348

Poutanen M, Montcharmont B and Vihko R (1992) 17β-hydroxysteroid dehydrogenase gene expression in human breast cancer cells: regulation of expression by a progestin. *Cancer Res* **52**: 290–294

Poutanen M, Meittinen M and Vihko R (1993) Differential estrogen substrate specificities for transiently expressed human placental 17beta-hydroxysteroid dehydrogenase and an endogeneous enzyme expressed in cultured COS-m6 cells. *Endocrinology* **133**: 2639–2644

Prudhomme JF, Malet C, Gompel A, Lalardrie JP, Ochoa P, Mauvais-Jarvis P and Kutten F (1984) 17β-hydroxysteroid dehydrogenase activity in human breast epithelial cell and fibroblast cultures. *Endocrinology* **114**: 1483–1489

Schneider J, Huh MM, Bradlow HL and Fishman J (1984) Antiestrogen action of 2-hydroxyestrone on MCF-7 human breast cancer cells. *J Biol Chem* **159**: 4840–4845

Siiteri PK, Simberg N and Murai J (1986) Estrogens and breast cancer. *Ann NY Acad Sci* **464**: 100–105

Strobl JS and Lippman ME (1979) Prolonged retention of estradiol by human breast cancer cells in tissue culture. *Cancer Res* **39**: 3319–3327

Suto A, Bradlow HL, Wong GYC, Osborne MP and Telang NT (1992) Persistent estrogen responsiveness of Ras oncogene-transformed mouse mammary epithelial cells. *Steroids* **57**: 262–268

Suto A, Bradlow HL, Wong GYC, Osborne MP and Telang NT (1993) Experimental down-regulation of intermediate biomarkers of carcinogenesis in mouse mammary epithelial cells. *Breast Cancer Res Treat* **27**: 193–202

Tait G, Newton CJ, Reed MJ and James VHT (1989) Multiple forms of 17beta-hydroxysteroid oxidoreductase in human breast tissue. *Mol Cell Endocrinol* **2**: 71–80

Telang NT (1996) Oncogenes, estradiol biotransformation and mammary carcinogenesis. *Ann N Y Acad Sci* **784**: 277–287

Telang NT, Banerjee MR, Iyer AP and Kundu AB (1979) Neoplastic transformation of epithelial cells in whole mammary gland in vitro. *Proc Natl Acad Sci USA* **76**: 5886–5890

Telang NT, Osborne MP, Sweterlitsch L and Narayanan R (1990) Neoplastic transformation of mouse mammary epithelial cells by deregulated *myc* expression. *Cell Regul* **1**: 863–872

Telang NT, Narayanan R, Bradlow HL and Osborne MP (1991) Coordinated expression of intermediate biomarkers for tumorigenic transformation in Ras-transfected mouse mammary epithelial cells. *Breast Cancer Res Treat* **18**: 155–163

Telang NT, Suto A, Wong GYC, Osborne MP and Bradlow HL (1992) Induction by estrogen metabolite 16alpha-hydroxy estrone of genotoxic damage and aberrant proliferation in mouse mammary epithelial cells. *J Natl Cancer Inst* **84**: 634–638

Telang NT, Katdare M, Bradlow HL and Osborne MP (1997) Estradiol metabolism: an endocrine biomarker for modulation of human mammary carcinogenesis. *Environ Health Perspect* **105**: 559–564

Vermeulen A, Deslypere JP, Paridaens R, Leclercq G, Roy F and Heuson JC (1986) Aromatase, 17β-hydroxysteroid dehydrogenase and intratissular sex hormone concentrations in cancerous and normal glandular breast tissue in postmenopausal women. *Eur J Cancer Clin Oncol* **22**: 515–525

Welsch CW (1985) Host factors affecting the growth of carcinogen-induced rat mammary carcinomas: a review and tribute to Charles Brenton Huggins. *Cancer Res* **45**: 3415–3443

Welsch CW (1987) Dietary retinoids and chemoprevention of mammary gland tumorigenesis. In *Cellular and Molecular Biology of Breast Cancer*. Medina D, Kidwell W, Heppner G and Anderson E (eds), pp. 495–508. Plenum Press: New York

*© Cancer Research Campaign 1998*